# Investigating the Cellular and Metabolic Responses of World-Class Canoeists Training: A Sportomics Approach

**DOI:** 10.3390/nu8110719

**Published:** 2016-11-11

**Authors:** Wagner Santos Coelho, Luis Viveiros de Castro, Elizabeth Deane, Alexandre Magno-França, Adriana Bassini, Luiz-Claudio Cameron

**Affiliations:** 1 Laboratory of Protein Biochemistry, Federal University of State of Rio de Janeiro, Rio de Janeiro 22290-240, Brazil; wagscoelho@hotmail.com (W.S.C.); luisviveirosdecastro@gmail.com (L.V.d.C.); bethcataldi@gmail.com (E.D.); amvfranca@gmail.com (A.M.-F.); abassini@me.com (A.B.); 2 Department of Biochemistry and Sportomics, Olympic Laboratory, Brazil Olympic Committee, Rio de Janeiro 22631-910, Brazil

**Keywords:** metabolism, biochemistry of exercise, ammonia, urate, exercise intensity biomarkers, physical stress response

## Abstract

(1) Background: We have been using the Sportomics approach to evaluate biochemical and hematological changes in response to exercise. The aim of this study was to evaluate the metabolic and hematologic responses of world-class canoeists during a training session; (2) Methods: Blood samples were taken at different points and analyzed for their hematological properties, activities of selected enzymes, hormones, and metabolites; (3) Results: Muscle stress biomarkers were elevated in response to exercise which correlated with modifications in the profile of white blood cells, where a leukocyte rise was observed after the canoe session. These results were accompanied by an increase in other exercise intensity parameters such as lactatemia and ammonemia. Adrenocorticotropic hormone and cortisol increased during the exercise sessions. The acute rise in both erythrocytes and white blood profile were probably due to muscle cell damage, rather than hepatocyte integrity impairment; (4) Conclusion: The cellular and metabolic responses found here, together with effective nutrition support, are crucial to understanding the effects of exercise in order to assist in the creation of new training and recovery planning. Also we show that Sportomics is a primal tool for training management and performance improvement, as well as to the understanding of metabolic response to exercise.

## 1. Introduction

Physical stress response due to a sport challenge is implicated in many metabolic modifications which affect the equilibrium of the biochemical internal environment [1,2]. This includes changes in the amount and kinetics of diverse biomarkers that are correlated with exercise intensity and muscle damage [3,4]. Some of these changes in metabolism can be assessed using blood as a biological matrix. For more than one decade, our group has dedicated research efforts towards understanding changes in metabolism using exercise as an induced-stress metabolic model [3,5,6,7,8,9,10,11,12,13,14,15,16]. The Sportomics approach targets metabolic and signaling molecule evaluations during either mimicked or real conditions faced in sports situations; it combines “-omics” technique with classic clinical laboratory analyses in order to understand sport-induced modifications [16]. These approaches represent a powerful tool to understand changes in physical and metabolic stress [17,18,19] and allow researchers to propose interventions in order to optimize athletes’ performance [5,20,21]. The approach is also a useful investigation tool for studying the effects of nutrition supplementation on physical training in different physiological or clinical conditions, such as type 2 diabetes mellitus [22]. Therefore, the analysis of world-class athletes in a field perspective allows us the possibility of understanding metabolic and signaling responses during high metabolic stress. Similar to a personalized-medicine approach, the Sportomics method allows us to better understand individual changes and to propose individualized interventions.

Several recent investigations have focused on the ammonemia changes resulting from a physical effort, which may be modified due to different causes [23,24,25]. Amino acids play a central metabolic role as an energetic source during exercise, which requires their deamination in order to be transduced into chemical energy. Increased muscle contraction rate also can contribute to changes in ammonemia, through adenosine monophosphate (AMP) deamination [26,27,28,29]. During intense or prolonged exercise, the reduced ability to resynthesize ATP promotes accumulation of ammonia and inosine monophosphate (IMP) which is metabolized to urate [30]. An intensity relationship has been proposed between ammonemia and exercise, as ammonia rapidly increases at intensities greater than 50%–60% of VO_2max_ [31,32]. However, ammonia production and release is not solely restricted to intense exercise. During prolonged (>1 h) submaximal exercise (60%–75% VO_2max_), ammonia could be produced through the breakdown of branched chain amino acid (BCAA) for additional energy provision [33,34,35].

Ammonia may cross the blood brain barrier causing neurotoxic effects including neuropsychiatric disorders, convulsion, and death [36], and may be implicated in central fatigue [25]. Therefore, ammonia accumulation may be avoided through a detoxification system. Humans convert ammonia to urea mainly in hepatocytes, and different cells can decrease ammonemia by synthesizing amino acids as a mechanism for further excretion of urea [9,11,37]. Therefore, an increase in urea levels reflects both AMP and amino acid deamination. On the other hand, urate is the final metabolite of the purine metabolism; hence, its measure can be stoichiometrically related to IMP deamination. Since urea and urate are, respectively, the final products of ammonia and purine metabolism, the study of the kinetics of those blood analytes leads to a better understanding of the metabolic pathways of ammonia origin and the response to exercise [37]. For this reason, our group has proposed nutritional and training interventions to promote metabolic adaptations in elite athletes to enhance their performance in training and competitions [3,10].

Canoeing has been featured as an Olympic sport since the Summer Olympic Games of 1936 in Berlin. Currently, men´s and women’s competitions cover distances of 200 m, 500 m, and 1000 m either solo, in pairs, or in crews of four. Canoeing contests are sprint events requiring sustained bursts of speed and power, leading to intense mechanical and metabolic stress. Little is known about these athletes’ metabolic responses during training sessions or competitions, therefore, the aim of this study was to evaluate four world-class canoeists during a training session through a Sportomics approach. As far as we know, this is the first metabolic investigation in the field, coming from our unique opportunity to investigate world-class athletes. This investigation will help enlighten us about the metabolism behavior in elite athletes.

## 2. Materials and Methods

This study assessed the metabolic response of four male world-class canoeists during a combined training session. All athletes were currently engaged in international elite competitions (including world championships, Pan-American, and Olympic games). During the trials, the athletes were instructed to maintain their typical hydration and food ingestion habits. Additionally, clinical evaluation, anthropometric measurements, and laboratory tests of collected blood samples were performed to assess health status. A Sportomics evaluation and analysis was performed to understand the metabolic effects of a training session. Subjects were fully instructed about the testing procedures and each signed a written informed consent. This study was conducted according to all procedures involving human subjects approved by the Ethics Committee for Human Research at the Federal University of the State of Rio de Janeiro (117/2007, renewed in 2011, 2013 and 2016) and met the requirements regulating research on human subjects (Health National Council, Brazil, 1996) the proper written informed consent was read and signed by the athletes.

### 2.1. Experimental Designs

**Figure 1:**
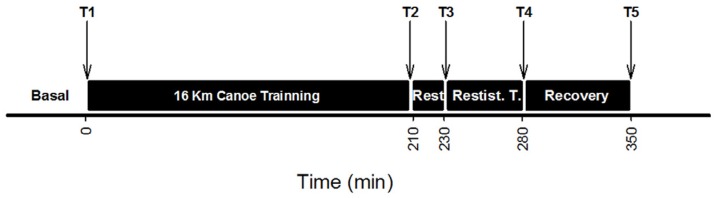
Experimental trial. Blood samples of the athletes were collected at the time points indicted in the Figure and as described in materials and methods.

After a regular warm up, the athletes were subjected to a training protocol that consisted of several canoe sprint bouts, with three minute intervals between each bout, covering different distances and intensities. The total distance totaled 16 kilometers. This first part of the protocol had a duration of 210 min followed by a rest period of 20 min during which they ingested a 500 mL beverage consisting of about 20% carbohydrate (short and medium absorption); 2% lipids; 5% proteins (casein and whey proteins). Next, they performed a weight lifting training session for 50 min focusing on exercises that recruit large muscle groups for both upper and lower body, followed by a 70 min of recovery. See the experimental trial depicted (Figure 1).

### 2.2. Blood Collection

Blood samples were collected following an antecubital vein puncture before (T1) and after (T2) the 16 km canoe training session; before (T3) and after (T4) the resistance training; and after the recovery period (T5) (Figure 1). Samples for hematological analysis assays were collected into tubes with K_2_-EDTA (Vacuette, Greiner Bio-One, Frickenhausen, Germany). White blood cell (total and differential), erythrocyte, and thrombocyte counts were measured in whole blood within a two-hour time frame after collection. Blood was immediately centrifuged to obtain either plasma or serum that was aliquoted, centrifuged (3000× *g*; 10 min; 4 °C), and stored in liquid nitrogen for later analysis (never more than eight hours). Samples were analyzed in duplicate or triplicate, when necessary, and measured against a standard curve with no less than five points.

### 2.3. Blood Analysis

A range of hematological and biochemical analyses was carried out totalizing around 100 analytes. The large amount of data generated was used in a non-target analysis linked to an ex-post facto study design. We chose near 20 analytes that could be relevant for our study of the athlete’s performance. Among others, our data set included a broad spectrum of metabolites and biomarkers related to different cellular and systemic signaling processes like inflammation and both muscle and hepatic injury.

Alanine aminotransferase (ALT), aspartate aminotransferase (AST), alkaline phosphatase (ALP), lactate dehydrogenase (LDH), γ-glutamyltransferase (γGT), creatine phosphokinase muscle-brain fraction (CKMB), creatine phosphokinase (CK), ammonia, urea, blood urea nitrogen (BUN), creatinine, urate, glucose, lactate, and 2-hydroxybutyrate were measured by the enzymatic kinetic method [38] in an automatic analyzer (ADVIA 1200—SIEMENS, Erlangen, Germany/Autolab 18 Boehringer Mannheim, Ingelheim am Rhein, Germany). Myoglobin was evaluated by the Hybridization Signal Amplification Method [39]. Albumin and total protein were assessed by electrophoretic analysis [40]. High-density lipoprotein (HDL), low-density lipoprotein (LDL), very-low-density lipoprotein (VLDL), total lipids, triacylglycerols (TG), and total cholesterol were assessed by the Chabrol & Charonnat method [41]. Amino acids were measured by high performance liquid chromatography (HPLC) [42]. CKMB-mass, insulin, adrenocorticotropic hormone (ACTH), and cortisol levels were assessed by chemiluminescence (Immulite 2000 Siemens, Erlangen, Germany) [43].

### 2.4. Statistical Analysis

Statistical analyses were performed using the software SigmaPlot 11.0 integrated with SigmaStat 3.5 packages (Systat, Santa Clara, CA, USA). Due to the nature of the experiment, including the similarity of subjects and the controlled experimental conditions (diet, sleep, training and major physical condition variables), the data were expressed as mean ± standard error (SEM). Data were normalized to pre-training results (T1) for clarity and analyzed by Analysis of Variance (ANOVA) using the condition and time as the repeated measured variables, which were confirmed using Tukey’s post hoc test. *p* < 0.05 was defined as the limit for statistically different mean values.

## 3. Results

Anthropometric characteristics of the individuals are presented on Table 1. Approximate averages of the values measured were as follows: 1.77 m of height, 82.9 kg of weight, 9.8 kg of fat weight, 73 kg of fat-free mass, 11.5% of body fat percentage, indicating that all tested individuals presented typical body composition, fat distribution, and weight profiles. We assessed the lipid profiles and serum protein levels of the individuals to characterize their nutritional status. As observed in Table 2, the assessed lipid profiles were in accordance with the healthy status of the general population. Table 3 presents the results regarding serum protein levels. Despite the fact that these data are considered normal values for the general population, it is worth noting that the assessed albuminemia was low considering a world-class team of athletes. Due to the lack of knowledge of world-class biomarker levels we chose to show all the data as a reference for future studies [3,5,10,21].

### 3.1. Muscle Stress Biomarkers

**Figure 2:**
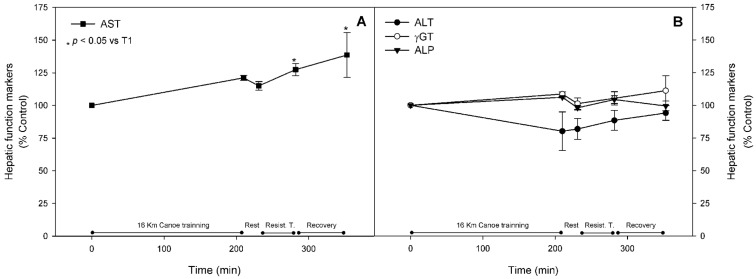
Hepatic injury biomarker. Aspartate aminotransferase (AST), alanine aminotransferase (ALT), γ-glutamyltransferase (γGT), and alkaline phosphatase (ALP) were measured as described in materials and methods and are represented as mean ± standard error of percentage values against control. * Indicates statistical difference against control values (*p* < 0.05). (**Panel A**) shows AST results and ALT, γGT, and ALP results are presented in (**Panel B**).

**Figure 3:**
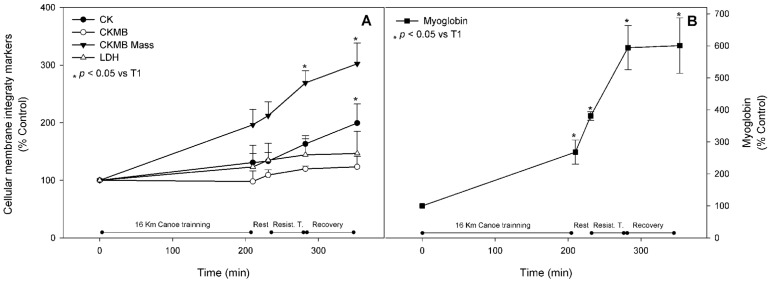
Cellular membrane integrity markers. Creatine phosphokinase (CK), creatine phosphokinase muscle-brain fraction (CKMB) activity and mass, lactate dehydrogenase (LDH) (**A**); and myoglobin (**B**) were measured as described in materials and methods and are represented as mean ± standard error of percentage values against control. * Indicates statistical difference against control values (*p* < 0.05).

Well-established metabolic stress biomarkers were assessed in order to characterize the training intensity of the proposed trial. Compared to basal levels, AST showed a statistically significant increase after the resistance training (T4) by 30%, and continued to increase by up to 40% after the recovery period. Other biomarkers, such as ALT, ALP, and γGT, did not change throughout the trial (Figure 2, panels A and B). CK activity in blood samples, a classic muscle injury marker, was significantly higher by approximately 60% at T4. Compared to the pre-exercise state, it kept increasing to nearly two-fold at T5 (Figure 3, panel A). Despite the fact that no significant change was observed in CKMB and LDH blood activity, the CKMB mass activity, a very specific muscle injury parameter, reached an increment of 170% at T4 and was up regulated by three-fold at T5 (Figure 3, panel A). Blood levels of myoglobin were significantly increased after the first session of exercise, with an increment of 170%, and kept increasing throughout the trial to reach six-fold values at T5 when compared to basal (Figure 3, panel B).

### 3.2. White Blood Cells

**Figure 4:**
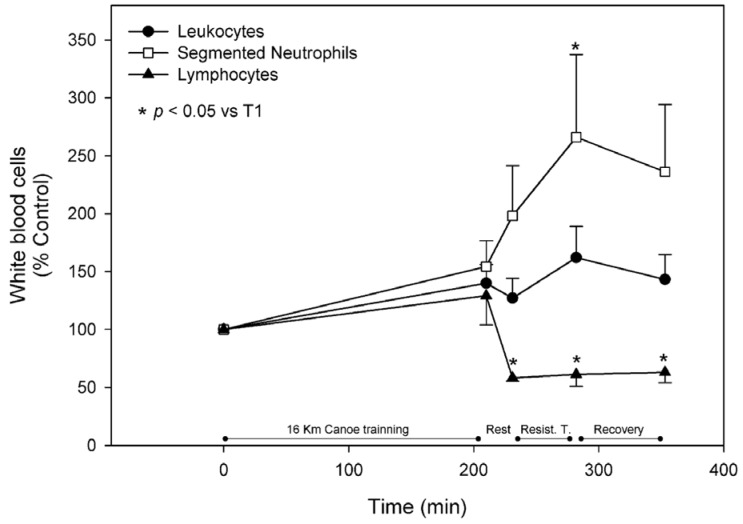
White blood cells. Leukocytes, segmented neuthrophils, and lymphocytes were measured as described in materials and methods and are represented as mean ± standard error of percentage values against control. * Indicates statistical difference against control values (*p* < 0.05).

**Figure 5:**
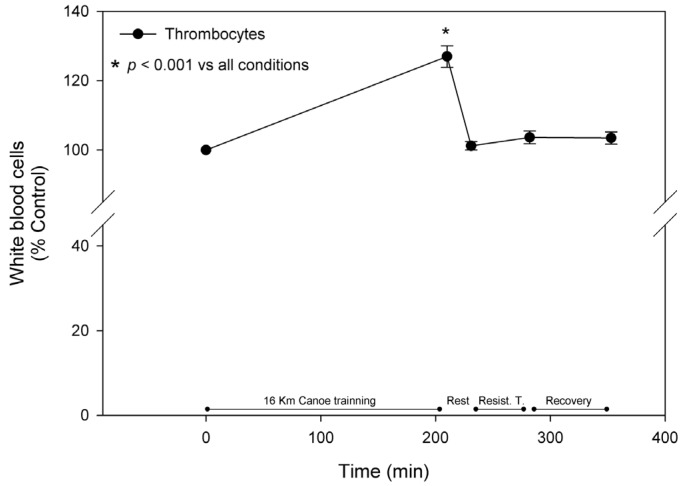
Thrombocytes. Thrombocyte levels were measured as described in materials and methods and are represented as mean ± standard error of percentage values against control. * Indicates statistical difference against any other condition (*p* < 0.05).

During the trial, blood leukocytes rose by 40.0% ± 16.1% and 62.1% ± 26.8% after the canoe (T2) and weight lifting (T4) sessions, respectively, showing a discrete decrease after recovery and reaching levels of 43.2% ± 21.5% higher than basal. These results were mainly due to the increment in the neutrophil count, which showed a significant increase by 54.3% ± 22.3% and 166.2% ± 71.4% after T2 and T4, respectively, and was still 136.0 ± 58.2 higher than basal levels after the recovery. Despite the slight increase after the canoe training, the levels of lymphocytes showed a significant decrease of approximately 40% after the 20 min rest between the training sessions and remained significantly lower until the end of the protocol (Figure 4). Eosinophils measurements tended to accompany the lymphocytes pattern, presenting an increment of approximately 30% at T2 followed by an acute reduction that remained until the trial was terminated. Monocytes acutely responded to exercise stress and the recovery periods, increasing by about 30% after both exercise sessions with a rapid restoration of the original values. Thrombocyte levels responded positively and significantly to the canoe training sessions; they increased 30% compared to the control at T2, acutely returned to basal levels after the 20 min rest prior following resistance training, and remained similar to the original value for the rest of the trial (Figure 5).

### 3.3. Branched Chain Amino Acids

**Figure 6:**
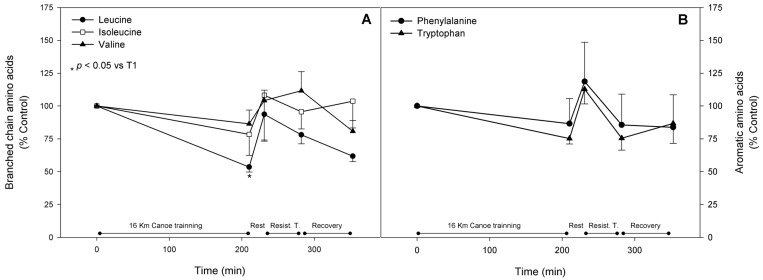
Branched chain (**A**) and aromatic (**B**) amino acids. Amino acid parameters were assessed as described in materials and methods and are represented as mean ± standard error of percentage values against control. * Indicates statistical difference against control values (*p* < 0.05).

Plasma branched chain amino acids (BCAA), which are important substrates either as metabolic fuel or as protein synthesis precursors, decreased right after both physical stimuli. Leucine showed the most prominent, significant decrease after the canoe training, reaching almost 50% of the basal value, and continued to decrease by approximately 22% after the resistance training. These decreases did not return to the original values, even after the recovery period. Both isoleucine and valine plasma concentrations seemed to be down regulated after canoe training, however, this decrease was not significantly different. After the 20 min rest between training sessions their levels returned to original values (Figure 6, panel A).

### 3.4. Aromatic Amino Acids

The three amino acids comprising the aromatic amino acids (AAA), phenylalanine, tryptophan and tyrosine, are ketoglucogenic amino acids that may be deviated to the gluconeogenic pathway in hepatocytes. Our results showed that these amino acids decreased in the range of 15%–25% after both the canoe and the weight lifting exercises (Figure 6, panel B).

### 3.5. Gluconeogenic Amino Acids

Many amino acids may serve as both substrates in anaplerotic reactions replenishing intermediates of the tricarboxylic cycle and as gluconeogenic substrates. Hence, many amino acids serve as energy sources in metabolic pathways. Interestingly, the plasma concentration of some of these amino acids increased right after the first exercise bout. Alanine showed a two-fold increment after the canoe training and also showed a slighter increment of approximately 20% at T4, after resistance training. Glutamate was up regulated by 63% at T2, but regained the original levels at T4. Ornithine plasma levels were enhanced by approximately 20% at T2. Methionine was elevated by approximately 86% after the canoe training session and decreased for the remainder of the trial. Taurine followed the methionine response, which is one of its precursors, rising 56% at T2. Glycine showed a later increase, with levels elevated by about 35% at T3 and with measurements similar to control at all other time points. On the other hand, arginine blood levels were down regulated to 60% of control values at T2 and 69% at T4. Glutamine presented a similar but slighter response, reaching 85% of basal values at T2 and returning to control levels thereafter. Lysine showed an approximately 30% decremented level at T2 when compared to control. Other assessed amino acids, such as asparagine, aspartate, serine, and threonine, did not fluctuate throughout the trial.

### 3.6. Metabolic Pathway Substrates, Intermediates, and Products

**Figure 7:**
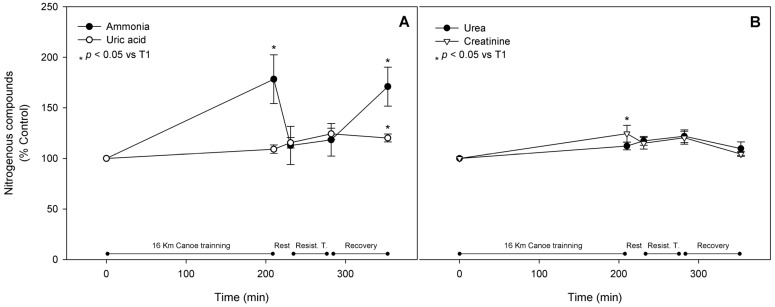
Nitrogenous compounds. Ammonia and uric acid are shown in (**Panel A**). Urea and creatinine fluctuations are presented in (**Panel B**). Nitrogenous compounds were evaluated as reported in materials and methods and are shown as mean ± standard error of percentage values against control. * Indicates statistical difference against control values (*p* < 0.05).

The metabolism of amino acids results in the production of nitrogen compounds, including ammonia; the increase in the levels of these compounds in the blood is tightly related to exercise intensity and duration. Ammonemia was significantly up regulated after the canoe training session by 78% and remained significantly enhanced (by about 71%) even after the recovery period. Urea blood level was slightly higher by about 21% at T4 and tended to reach normal values after the recovery period. Urea concentration may reflect total ammonia excretion. IMP production is correlated to urate appearance in the blood, which is the final product of purine catabolism. Urate blood concentration rose significantly in blood by 24% and 20% at T4 and T5, respectively, when compared to the control. Blood levels of creatinine, a muscle damage indicator that may also suggest hemoconcentration alteration, responded acutely to both exercise sessions, augmenting significantly by 24% at T2. This was followed by a restoration of control values at T3, then a significant enhancement right after resistance training by 20% when compared to the control, and then a return to normal values at the end of the recovery period (Figure 7). BUN concentration remained unchanged throughout the trial.

**Figure 8:**
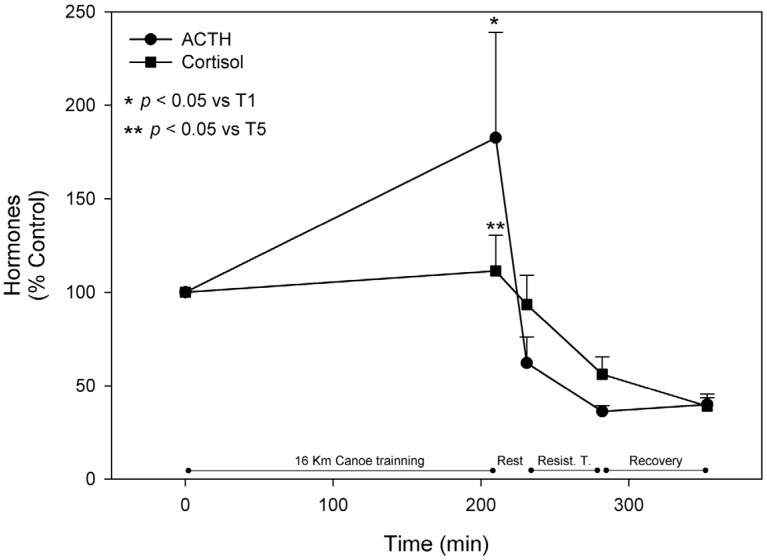
Hypothalamic-pituitary adrenal axis hormones. Adrenocorticotropic hormone (ACTH) and cortisol were measured as described in materials and methods and are represented as mean ± standard error of percentage values against control. * Indicates statistical difference against control values (*p* < 0.05). ** Indicates statistical significance when compared to T5 values (*p* < 0.05).

Serum glucose, insulin, ACTH, and cortisol fluctuations during exercise are also related to the destinations of amino acid metabolites and, therefore, were measured. Due to the stress caused by the canoe training session, the hypothalamus activated the production of Corticotropin Releasing Hormone (CRH), which in turn stimulated the anterior pituitary gland to produce ACTH, and then the adrenal gland to produce cortisol. While ACTH serum levels increased by 82% at T2, cortisol rose only 12% in comparison to initial levels. After exercise, the HPA axis was suppressed, and blood levels of both hormones diminished significantly when ACTH reached values of 36% at T4, and measured cortisol was 39% at T5 when compared to T2 (Figure 8).

**Figure 9:**
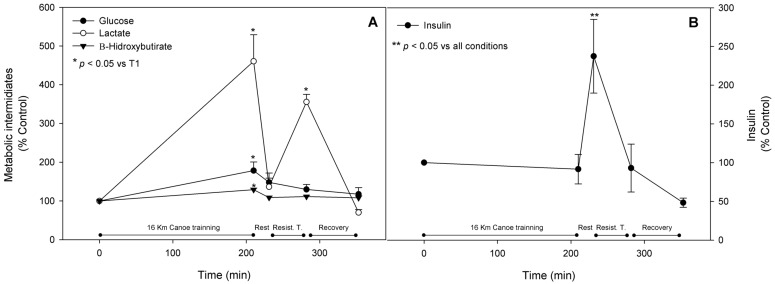
Metabolic intermediates and Insulin. Glucose, lactate, 2-hidroxybutirate (A), and insulin (B) were assessed as described in materials and methods and are represented as mean ± standard error of percentage values against control. * Indicates statistical difference against control values (*p* < 0.05). ** Indicates statistical significance when compared to all conditions (*p* < 0.05).

Insulin presented a slight decrease in response to the canoe exercise by about 10% and a significant increase of 137% after the 20 min of rest due to the food and hydro-electrolyte reposition; it returned to the approximate basal levels after the resistance training session and presented a drop by 50% of the original concentration. Glycemia was significantly enhanced by 78% after the canoe bout and tended to decrease progressively throughout the trial while maintaining its blood level slightly higher than basal. The ketone body 2-hydroxybutyrate blood levels were also significantly augmented after the canoe bout by 29%, followed by a slighter increment of 11% after the second exercise training session (Figure 9, panels A and B). It is well known that the lactate blood levels increase according to the exercise intensity. Our results showed a significant increment of 360% and 255% after the canoe and resistance training sessions, respectively (Figure 9, panel A).

## 4. Discussion

Canoeing competitions are sprint events requiring sustained bursts of speed and power, leading to intense biochemical and metabolic stress. Many metabolites produced due to physical effort may be implicated in fatigue and impairment of physical performance, hence, the understanding of these responses is crucial to upgrade training sessions and optimize athlete performance. Here, we applied a Sportomics approach to study and evaluate different metabolic and cellular responses during a training session of world-class canoe athletes. Sportomics can help us in the understanding of the metabolism and signaling events that occur in response to exercise and allow us to perform interventions increasing both metabolic and sportive performances [10,16,19].

We measured serum levels of known exercise intensity biomarkers to characterize the applied training protocol magnitude and assure the possibility of correlating exercise intensity to the metabolic responses. Exercise intensity can be inferred by increases in plasma levels of CK, CKMB mass, CKMB, and LDH [3,44,45,46]. Several studies have investigated the increase in CK in response to exercise [3,5,10,21]. In this study, CK and CKMB mass blood levels increased continuously throughout the exercise trial, rising significantly at T4 and T5 when compared to basal levels. This suggests that the exercise stress and duration represented enough stimuli to cause such changes; a similar result was described by Siegel et al. [47]. Despite these observations, CKMB levels did not elevate significantly at any time point throughout the exercise session. However, it is worth noting that the blood basal levels of this enzyme were higher than expected due to exercise accumulation along the regular training season of the athletes, and this fact may have limited the furthest increment along the executed protocol. Additionally, immune assays show more analytical sensitivity when compared to enzymatic activity measurements. Therefore, it is necessary to separate immune assays from enzymatic assays. It is important to emphasize that most protocols measure the enzymatic activity of CK (also LDH and others) as a way to understand its increase or decline in response to exercise. These enzymes are also being subjected to blood environment changes that can lead to an increase in the specific activity (i.e., the ability to an enzyme to catalyze a reaction in a given unit of time), so it is our understanding that the preferable way to measure muscle cellular injury is to use a direct measurement of the enzyme content using immunological quantitative methods (such as ELISA or Western blot or mass spectrometry). For us, this is an important statement because we believe that researchers should carefully analyze the results of any increase of enzymes during exercise by the way of enzymatic activity.

Blood levels of LDH also remained constant throughout the exercise trial, and this result is in accordance with other studies which have shown a classic delayed response of LDH blood levels to strenuous exercise [48,49,50]. Myoglobin release from muscle to blood is a well-known biochemical marker of muscle injury [51]. In our study, myoglobin significantly increased due to the exercise stress, confirming skeletal muscle damage induced by the exercise protocol. This confirms the CK findings and corroborates our interpretation concerning the difference in the CK and CKMB increase.

Classical biochemical hepatic damage markers such as AST, ALT, ALP, and γGT can be increased as a result of liver and/or muscle injury after strenuous exercise [52,53]. In the present study, the only enzyme that presented a significant increase throughout the protocol was AST, while the serum level of the other enzymes remained unchanged throughout the exercise. Since these enzymes are also present in muscles, the origin of this increase could not be differentiated between muscle or liver cell disruption. However, we have recently proposed that muscle damage can be distinguished from liver damage by using the more specific liver biomarkers such as ALT and γGT [5], which remained constant in the present study. These data together suggest that the increases of these proteins are more likely from muscle rather than liver cellular injury.

White blood cell counts increase after many types of exercise and the release of neutrophils is directly correlated with exercise intensity and duration [3,17,18]. In this study, we reported an increment in total leukocytes in response to both exercise sessions, which can be attributed to the release of neutrophils. Interestingly, after a discrete increment in lymphocyte levels at T2, it returned to basal values and continued decreasing reaching a significant reduction between the training sessions. Previous studies indicated that the response of white blood cells to exercise is dependent on both cytokine and myokines modulation [54,55,56]. Taken together these results may suggest that white blood cell mobilization is due not to a non-specific exercise-induced spleen release, but rather to a specific signal. Additionally, we reported an increase in platelet count without any change in erythrocyte count, indicating that this effect occurs in a spleen-independent manner. As previously suggested, these results may indicate that both thrombocytosis and leucocytosis observed during the exercise bout are induced by either muscle cell damage or differential cell signaling [3,18].

Muscle cell damage is known to stimulate immune cell mobilization to the bloodstream and migration to muscle tissue [5]. Exercise has been proposed to be a physiological way to modulate immunity; while acute severe exercise usually impedes immunity, chronic moderate exercise improves it [57,58]. Although the evidence to support these concepts is inconclusive, it supports the idea that exercise-induced immune suppression increases susceptibility to symptoms of infection, particularly around time of competition [59]. Moreover, metabolic stress is correlated with exercise induced white blood cell response, as carbohydrate supplementation and availability have been proposed to affect neutrophil count after intense exercise [60,61,62]. We previously described that a combination of training, rest and nutritional intervention could have an important impact in amino acid availability, muscle cellular injury, and immune response in another world-class athlete [10]. Therefore, the immune responses reported here may be directly correlated with the alterations in the nutritional status and metabolic availability as observed during the present experimental trial.

In this sense, it is important to maintain plasma level amino acids during training sessions, since many amino acids serve both as anabolic and energetic precursors. In addition, it has been proposed that blood fluctuations in the concentration of BCAAs may affect its ratio in the brain [63]. In our study, the levels of many amino acids presented a blood concentration decrease during the sport trial. Leucine showed the most important decrease after the canoe training session. Isoleucine and valine concentration also decreased in a smaller range. Aromatic amino acids, which are generally metabolized in the liver, were slightly consumed during the canoe exercise session; similar results were described before [10]. Glutamine levels presented a similar response; they decreased after the canoe trial and were restored after the recovery period. This could be the result of two processes: glutamine exportation from muscle to decrease its ammonia levels; and the use of glutamine as both a gluconeogenic substrate and a urea cycle feeder in the liver. On the other hand, alanine was up regulated after the first exercise bout, showing a two-fold increment at T2. This response may be attributed to a metabolic attempt to offer gluconeogenic substrates for further oxidation. The depletion of glycogen storage is related to exercise intensity, duration, and nutritional status, which in turn may increase the use of amino acids as energy substrates, thereby increasing ammonia and the production of other nitrogen compounds [64]. Both glutamine and alanine are anaplerotic and gluconeogenic substrates and contribute to ATP and glucose synthesis. The ergogenic properties of glutamine have been extensively studied [6,62,65], and we have recently reported the metabolic effects of alanine in comparison to long-term glutamine supplementation during an intermittent exercise protocol. Long term administration of glutamine is capable of reducing ammonia production during intermittent exercise, hence, it is postulated to be a protector against an increase in blood ammonia in an exercise intensity-dependent manner [21].

Many studies have indicated that ammonia is a useful physiological marker of prolonged intense exercise, and its appearance in blood is positively correlated with exercise intensity [1,10,30]. High ammonemia can be toxic to both muscles and the central nervous system (CNS). Such changes are believed to contribute to the disturbances in neuropsychological function and motor control deficits and are also observed in patients with cirrhosis and, therefore, could induce central and peripheral fatigue [25,60,66]. Therefore, measuring ammonia production during a sport session may represent an important tool to control exercise intensity and to understand the metabolic response of a given athlete. The canoe athletes experienced an increase in their blood ammonia levels during the exercise trial due to both stimuli, which remained up regulated even after the recovery period. This effect was followed by an increase in other measured nitrogenous compounds, such as urea, urate and creatinine. These responses may have occurred as a result of an increased demand for ATP by muscle contraction, leading to adenosine monophosphate (AMP) deamination and, subsequently, the production of ammonia and urate [26,27,28,29]. Many studies have shown that ammonia production and release represents the exercise effort intensity, rapidly increasing in intensities greater than 50%–60% of VO_2max_ up to maximal exhaustion [31,32]. Ammonia plasma concentration is also up regulated during prolonged (greater than one hour) submaximal exercise (60%–75% VO_2max_). In these conditions, ammonia could be produced in increasing amounts through the breakdown of branched chain amino acid (BCAA) prior to oxidation for additional energy provision [33,34,35].

The response of the other nitrogen metabolites may shed light on understanding the protein and amino acid oxidation response during exercise. Urea and urate blood concentration is indirectly correlated with the myokinase (adenylate kinase, ADK) contribution to ATP synthesis. Under a resting physiological state, approximately 90% of the skeletal muscle adenosine monophosphate deaminase (AMPD) is in a sarcoplasmic position and in an inactive form. However, a significant change occurs as intense muscle contraction begin, when approximately 50%–60% of AMPD becomes bound to the myofibrils [28]. Binding of the enzyme increases its activity causing an increased rate of degradation of AMP to IMP. This is correlated with the appearance of urate in the blood which is a final metabolite of purine metabolism [30]. This increased breakdown of AMP will affect the equilibrium of the ADK reaction by creating additional ATP from ADP to increase the cellular energy charge and maintain contractions under conditions of increasing stress [29]. During intense exercise, when AMP production and deamination are high, ADP levels also increase as utilization of ATP exceeds re-phosphorylation [67]. Therefore, any strategies, such as diet adequacies and supplementations, to protect against hyperammonemia or an increment of any nitrogenous compound could enhance physical performance or prevent CNS injuries, as previously reported by our group [6,10,21].

During the canoe trial, glycemia rose significantly, which may be a result of the HPA axis activation. Exercise is known to be a potent activator of this endocrine system, resulting in the release of ACTH, as confirmed here. This ultimately culminates with glucocorticoids production and release into blood circulation, which may lead to gluconeogenesis activation and promotion of an adrenergic stimulus, providing glucose to blood from hepatic glycogenolysis [68,69,70]. Nevertheless, afferent neural feedback signals from contracting muscle and feedback signals mediated via the blood stream can stimulate glucose production to maintain glycemia. Therefore, central mechanisms coupled with the degree of motor center activity can be responsible for part of the increase in glucose mobilization, especially during intense exercise where hepatic glucose release exceeds peripheral glucose uptake, and plasma glucose rises [71]. Furthermore, cortisol is implicated in exercise induced lymphocyte apoptosis, via glucocorticoid dependent-pathways [72], which might affect immune function and protect the organism from an overreaction of the immune system in the face of exercise-induced muscle damage [73].

Hepatic glucose production increases during exercise, to cope with the augmented demand, as a product of liver glycogenolysis and gluconeogenesis. Whereas the former predominates during high intensity exercise, the latter contributes substantially with prolonged exercise and the concomitant decline in liver glycogen stores and with increased gluconeogenic precursor supply. In fact, it has been postulated that the increase in glucose production with exercise intensity in healthy subjects can be entirely attributed to increases in net hepatic glycogenolysis [74]. This pathway is also supported by our data. On the other hand, a decline in plasma insulin is important for the rise in glucose production during exercise [71], due to the fact that insulinemia tends to decrease in response to prolonged exercise, with a more pronounced effect on athletes than untrained individuals [75], which is in agreement with the results reported here.

## 5. Conclusions

The data presented here allow us to consider hormonal, metabolic, and signaling response together with the knowledge of nutrition and training environment. This combined information permits a better understanding of the individual responses of exercise and sport stress. Our group developed the concept of Sportomics with a focus on bridging the same existent gap between translational and personalized medicine [76]. As stated by Liebman et al. [77], the workflow bench to bedside approach is being refined in the face of a new bedside-bench-bedside approach. Sportomics is useful to evaluate the unprecedented kinetics of some metabolites [3,5,10,11,13,14,16,37,78] and to shed light on the importance of in-field metabolic analyses to the understanding of the inter-individual response to exercise. Besides an effective nutritional support, collecting physiological data during training and competition can provide important information about an athlete’s clinical condition, bringing strategies to modify metabolism during exercise as well as supporting coaches to prescribe their sessions and recovery time. Therefore, and due to the uniqueness of this study, we believe Sportomics is a primal tool for training management and performance improvement, as well as for preserving health and increasing the quality of life of athletes.

## Figures and Tables

**Table 1:** Anthropometric parameters of the athletes were measured and are shown here as mean ± standard error.

Anthropometry	
Height (m)	1.77 ± 0.02
Weight (Kg)	82.9 ± 5.0
Fat weight (Kg)	9.8 ± 2.4
Fat-free mass (Kg)	73.0 ± 2.6
Fat percentage (%)	11.5 ± 2.0
BMI (Kg/m^2^)	26.2 ± 1.2

**Table 2:** Lipid panel values–high-density lipoprotein (HDL); low-density lipoprotein (LDL); very low-density lipoprotein (VLDL)-were assessed as described in materials and methods and are shown here as mean ± standard error.

Lipid Panel (mg/dL)	
Serum cholesterol	173.6 ± 24.2
Serum triacylglycerol	83.3 ± 20.0
HDL	55.3 ± 5.0
LDL	95.0 ± 26.5
VLDL	16.6 ± 3.8
Cholesterol/HDL ratio	3.2 ± 0.7
LDL/HDL ratio	1.9 ± 0.6
Non cholesterol lipids	118.3 ± 28.9

**Table 3:** Protein fractions were assessed as described in materials and methods and are shown here as mean ± standard error.

Protein Fractions (g/dL)	
Total proteins	6.7 ± 0.12
Albumin	2.7 ± 1.23
α-1 globulin	0.4 ± 0.14
α-2 globulin	0.5 ± 0.05
β-1 globulin	0.4 ± 0.02
β-2 globulin	0.2 ± 0.003
γ globulin	1.2 ± 0.12
